# Establishment of a large panel of patient-derived preclinical models of human renal cell carcinoma

**DOI:** 10.18632/oncotarget.10659

**Published:** 2016-07-18

**Authors:** Hervé Lang, Claire Béraud, Audrey Bethry, Sabrina Danilin, Véronique Lindner, Catherine Coquard, Sylvie Rothhut, Thierry Massfelder

**Affiliations:** ^1^ Department of Urology, Hôpitaux Universitaires de Strasbourg, Nouvel Hôpital Civil, Strasbourg, 67091 France; ^2^ UROLEAD SAS, School of Medicine, Strasbourg, 67085 France; ^3^ INSERM U1113, Section of Cell Signalisation and Communication in Kidney and Prostate Cancer, University of Strasbourg, School of Medicine, Fédération de Médecine Translationnelle de Strasbourg (FMTS), Strasbourg, 67085 France; ^4^ Department of Pathology, Hôpitaux Universitaires de Strasbourg, Hôpital de Strasbourg-Hautepierre, Strasbourg, 67200 France

**Keywords:** renal cell carcinoma, human tumors, patient-derived xenograft models

## Abstract

The objective of the present work was to establish a large panel of preclinical models of human renal cell carcinoma (RCC) directly from patients, faithfully reproducing the biological features of the original tumor. RCC tissues (all stages/subtypes) were collected for 8 years from 336 patients undergoing surgery, xenografted subcutaneously in nude mice, and serially passaged into new mice up to 13 passages. Tissue samples from the primary tumor and tumors grown in mice through passages were analyzed for biological tissue stability by histopathology, mRNA profiling, von Hippel-Lindau gene sequencing, STR fingerprinting, growth characteristics and response to current therapies. Metastatic models were also established by orthotopic implantation and analyzed by imagery. We established a large panel of 30 RCC models (passage > 3, 8.9% success rate). High tumor take rate was associated with high stage and grade. Histopathologic, molecular and genetic characteristics were preserved between original tumors and case-matched xenografts. The models reproduced the sensitivity to targeted therapies observed in the clinic. Overall, these models constitute an invaluable tool for the clinical design of efficient therapies, the identification of predictive biomarkers and translational research.

## INTRODUCTION

Renal cell carcinoma (RCC) is the most lethal urologic tumor and the sixth leading cause of cancer deaths in Western countries. Each year, around 340,000 patients are diagnosed with this malignancy resulting in approximately 150,000 deaths, and its incidence is increasing steadily [1]. RCC is resistant to radiotherapy and systemic therapies including the current targeted therapies. Although current therapies, including sunitinib, sorafenib and everolimus, have proven beneficial in treating RCC, complete response remains a rare event [2]. The lack of validated biomarkers restricts our ability to tailor specific drugs to patients and might be considered as the most important barrier for a better clinical outcome.

RCC tumors consist of several histological subtypes, including clear cell (CCC, ~75%), papillary (~12%), chromophobe (~4%), collecting duct (~1%) and unclassified (~4%) carcinomas [2]. Models of human cancer in mouse or rat are critical (i) for a better understanding of the tumor pathobiology, invasion and resistance, (ii) to define new therapeutic options, (iii) to identify predictive biomarkers guiding adequate therapy and (iv) to identify prognostic and diagnostic biomarkers. It is however essential that animal model mimics as closely as possible the heterogeneity of the original tumors to reach these goals.

Hereditary RCC occurs in Eker rats that are heterozygous for an insertion mutation in the rat homologue of the tuberous sclerosis complex 2 (*Tsc2*, encoding tuberin), a tumor suppressor gene that renders heterozygous mutants highly susceptible to renal carcinogens [3, 4]. This model, in which the incidence of RCC in gene carriers approaches 100% by 1 year of age, has been used to study the molecular pathways of renal tubular epithelial carcinogenesis, but despite its significance for studying some of the genetic alterations occurring during renal tumorigenesis, it represents mostly chromophobe RCC and benign oncocytoma arising from the collecting duct and not from the proximal tubule as for CCC [5]. There are no transgenic models of RCC despite the attempt of some investigators to develop such mice by interfering with the expression of von Hippel-Lindau (VHL) tumor suppressor proteins [6], that are part of the machinery leading to HIF factors degradation, HIF [7] or Pax2 transcription factors [8]. RCC models currently available are based on the subcutaneous (generally non-invasive model) or orthotopic (invasive model) implantation of human RCC cell lines into nude mice [9–11]. These models suffer from various limitations including that (i) they are clonal cell lines that do not recapitulate the heterogeneity of the tumors found *in situ*, (ii) the number of available and characterized cell lines is limited and (iii) cancer cells cultured *in vitro* are known to acquire genetic variability not found in the original tumors and to be sentitive to all therapeutic compounds [12–14], a behaviour not found in the *in vivo* environment.

To date, the most accurate models are patient-derived tumor xenografts (PDX) resulting from the implantation of viable cancer tissues into nude mice, as it has been shown for various cancer types, including bladder [15], breast [16], pancreatic [17], lung [18], ovarian [19], colon [20], liver [21] cancers and melanoma [22]. These models reflect the heterogeneity of the original tumors and allow tumor-stroma interactions found in tumors *in situ* that cannot be recapitulated by *in vitro* experiments. Few studies using a limited panel of patients show that such approaches are suitable to develop patient-derived RCC models in nude mice [23–34].

In the current study, we describe the development of a large panel of well-characterized patient-derived RCC models based on subcutaneous implantation of freshly harvested tumors. Our results show that these models reproduce the sensitivity to targeted therapies observed in the clinic and that they very closely mimic human RCC, providing valuable opportunities to increase our knowledge of kidney tumorigenesis.

## RESULTS

### Tumor implantation and growth characteristics

During the last 8 years, 336 RCC tumors were obtained directly from patients who underwent either partial or radical nephrectomy (Table 1). Eligibility criteria were based on preoperative imaging studies and included tumors of all subtypes and stages, multifocal, bilateral or, regional.

**Table 1:** Patients, Tumor and PDX characteristics

		Original Tumor n (%)	PDX models n (%)
**Age**	< 60 (32-58 ; 50.4±2.7)	131 (39)	10 (33.3)
	≥ 60 (60-86 ; 70.1±1.6)	205 (61)	20 (66.7)
**Sex**	Female	138 (41)	8 (27)
	Male	198 (59)	22 (73)
**RCC subtype**	CCC	262 (78)	24 (80)
	Papillary RCC	26 (7.7)	1 (3.3)
	Oncocytoma	21 (6.3)	-
	Chromophobe RCC	18 (5.4)	1 (3.3)
	Composite RCC	5 (1.5)	2 (6.7)
	Medullary RCC	2 (0.6)	1 (3.3)
	Unclassified RCC	2 (0.6)	1 (3.3)
**pT stage**	pT1	5 (1.6)	-
	pT1a	109 (35.5)	1 (3.3)
	pT1b	60 (19.5)	6 (20)
	pT2	14 (4.5)	-
	pT2a	7 (2.3)	-
	pT2b	2 (0.7)	-
	pT3	10 (3.3)	2 (6.7)
	pT3a	42 (13.7)	5 (16.7)
	pT3b	45 (14.7)	10 (33.3)
	pT3c	3 (1)	3 (10)
	pT4	10 (3.3)	3 (10)
**Furhman grade**	1	20 (6.8)	-
	2	142 (48.3)	4 (13.8)
	3	96 (32.7)	12 (41.4)
	4	36 (12.2)	13 (44.8)

**Figure 1:**
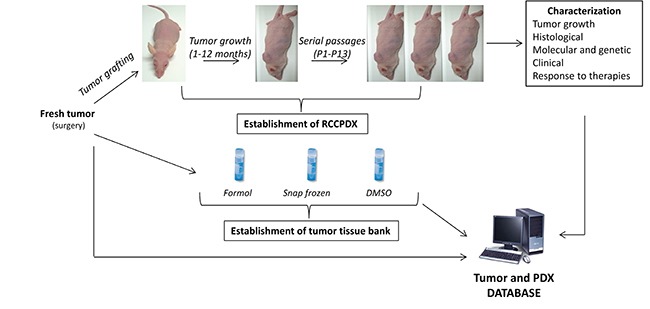
Schematization of the RCCPDX development processes They include the establishment of the PDXs, their characterization at the different indicated levels and the establishment of tumor tissue bank, all data forming the tumor and PDX database.

Most patients were males (59%) and their age ranged between 32 to 86 years (Table 1). Over 90% were renal cell carcinoma and 78% were of the clear cell type (Table 1). About 50% of the RCC were of high grade and sarcomatoid elements were found in 13% of cases. Thirty tumor grafts were passaged at least three times (P3) in mice (take rate 8.9%) and these are referred to as models RCCPDX1 to RCCPDX30 depending on the time of establishment (Table 2). The developing process is presented in Figure 1.

**Table 2:** RCCPDX characteristics

RCCPDX ID	Gender	Age at diagnosis	Year of first engraftment in mouse	RCC subtype	pTNM stage	Fuhrman grade	Sarcomatoid features (%)
RCCPDX1	M	69	2007	CCC	pT3bN2	3	
RCCPDX2	M	86	2007	CCC	pT3bNx	4	<1%
RCCPDX3	M	70	2008	CCC	pT3bN1	3	20%
RCCPDX4	F	60	2008	CCC	pT1bNx	2	
RCCPDX5	M	70	2008	CCC	pT3bN0	3	
RCCPDX6	M	58	2008	Composite RCC	pT3cN2	3	20%
RCCPDX7	M	61	2008	CCC	pT3bN0	3	
RCCPDX8	M	75	2009	CCC	pT1bNx	4	15%
RCCPDX9	M	60	2009	CCC	pT1bN0M1	4	30%
RCCPDX10	F	53	2009	Chromophobe RCC	pT4N0	4	80%
RCCPDX11	F	39	2009	CCC	pT3bN1	4	50%
RCCPDX12	F	65	2009	CCC	pT1bNx	2	
RCCPDX13	M	57	2009	CCC	pT3b	4	
RCCPDX14	M	74	2010	CCC	pT3aNxMx	3	
RCCPDX15	M	62	2010	CCC	pT3cN2Mx	4	15%
RCCPDX16	M	74	2010	CCC	pT3bN0Mx	2	
RCCPDX17	M	80	2010	CCC	pT1b	2	
RCCPDX18	F	76	2010	CCC	pT4N2	4	20%
RCCPDX19	M	57	2011	CCC	pT3bM1	4	20%
RCCPDX20	M	51	2011	Composite RCC	pT3bN1	4	5%
RCCPDX21	M	49	2011	CCC	pT3aN2M1	3	40%
RCCPDX22	M	72	2011	CCC	pT3aN2	3	
RCCPDX23	M	66	2011	CCC	pT1a	3	
RCCPDX24	F	75	2011	Unclassified RCC	pT4Nx	4	100%
RCCPDX25	F	69	2012	CCC	pT3cN0	3	
RCCPDX26	M	52	2012	CCC	pT3N0	3	
RCCPDX27	M	32	2012	Medullary RCC	pT3	/	
RCCPDX28	F	64	2012	CCC	pT1bNx	4	
RCCPDX29	M	74	2013	Papillary RCC	pT3aN2	3	
RCCPDX30	M	56	2014	CCC	pT3aNx	4	

**Figure 2:**
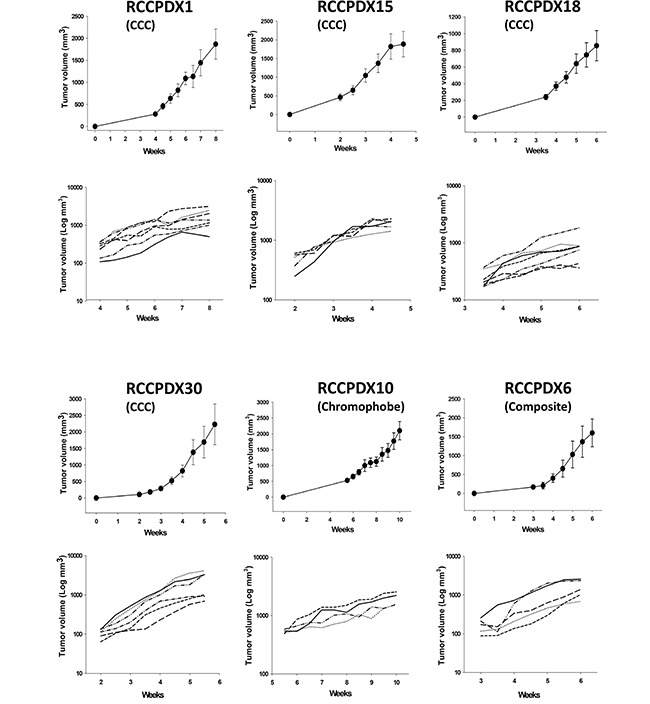
*In vivo* growth curves of 6 RCCPDX tumors after implantation in nude mice Curves are shown for 4 RCCPDX of the CCC subtype, 1 RCCPDX of the chromophobe subtype and 1 composite RCCPDX. Top graph, growth curve for each RCCPDX expressed with linear Y scale axis; bottom graph, growth curve for each individual mouse expressed with Y axis in Log scale showing the stable behavior of tumor growth. X-axis: days after implantation; Y-axis: tumor volume in mm^3^. n=4 to 7. Note: For RCCPDX1, RCCPDX15 and RCCPDX30, mice were euthanized when tumor volume reached the ethical 2000 mm^3^.

We noticed that tumor stage, high Fuhrman grade as well as sarcomatoid differentiation were associated with higher engraftment. We obtained a 4.0% success rate at pT1 stage (7 RCCPDX models from xenografting 174 tumors) vs. a 20% success rate at pT3 stage (RCCPDX models from xenografting 102 tumors). In our study, we chose to xenograft all RCC tumors operated at the New Hospital Civil of Strasbourg, in order to have a panel of RCCPDX models covering all stages. Thus there were no ineligibility criteria for the tumors we implanted. Concerning Fuhrman grade, for grade 1, the xenograft success rate was 0% (O RCCPDX models from xenografting 20 tumors); for grade 2, the success rate was 2.8% (4 RCCPDX models from xenografting 142 tumors); for grade 3, the success rate was 12.5% (12 RCCPDX models from xenografting 96 tumors) and from grade 4, the success rate was 36.1% (13 RCCPDX models from xenografting 36 tumors). Thus, for low grade (1 + 2), the success rate was 2.5% (4 RCCPDX models from xenografting 162 tumors) and for high grade (3 + 4), the success rate was 18.9% (25 RCCPDX models from xenografting 132 tumors). For sarcomatoid differentiation, 13 PDX were developed from 44 tumors, i.e. ~ 30% (Table 1). There were no other tumor parameters influencing this rate, among the ones studied. In addition, the average latency period for the first growth in mice was variable, ranging from 1 to 12 months. Again, there was no tumor parameter influencing this data. Tumor growth was assessed in some models and was dependent on the RCCPDX model but was quite similar from mouse to mouse (Figure 2) and from passage to passage (data not shown). All models were free of viruses and pathogens (data not shown).

### Histologic, molecular and genetic stability of the models

**Figure 3:**
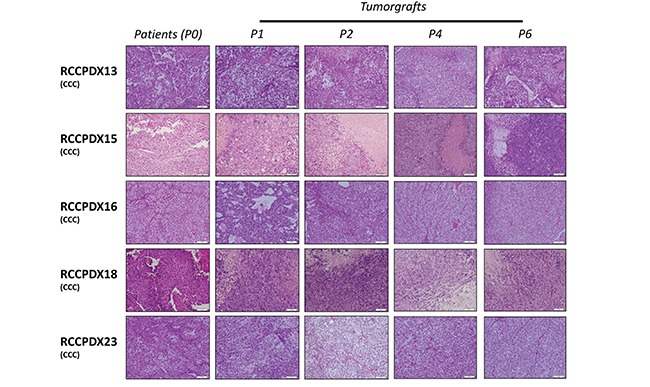
Histologic characterization of RCCPDX models Representative hematoxylin and eosin sections (x 400) of 5 RCCPDX tumors of the CCC subtype comparing the original patient tumor (P0) to 4 passages in mice. P1, first xenograft in mice; P2, second xenograft in mice; P4, fourth xenograft in mice and P6, sixth xenograft in mice.

A very important requirement for PDX models is that they should keep the histologic, molecular and genetic characteristics of the patient's tumor from which they derived to have preclinical and clinical relevance. We performed H&E staining on all RCCPDX models at P0 (primary tumor) and at the different subsequent passages in mice, as indicated (Figure 3 and Table 3). Histopathology analysis of all models was performed by an experienced pathologist specialized in uropathology, and showed that RCCPDX models retained the histology features of the parental tumor, including cancer subtype, stage, cytological shape, and Fuhrman grade.

**Table 3:** Histological analysis of RCCPDX models and corresponding original tumor

ID	Passage	Histology	GRADE	Architecture Cytoplasmic features
**RCCPDX1**	0	clear cell	3	acidophilic / tubular / acinar
	3	clear cell	4	clear acidophilic
	4	clear cell	3	acidophilic / clear
	5	clear cell with 80% sarcomatoid	4	acidophilic / spindle cell
	6	clear cell with 80% sarcomatoid	4	acidophilic / spindle cell
	7	clear cell with 70% sarcomatoid	4	acidophilic / spindle cell
**RCCPDX2**	0	poorly differenciated carcinoma with < 1% sarcomatoid	4	acidophilic
	1	poorly differenciated carcinoma with < 1% sarcomatoid	4	acidophilic / clear
	2	poorly differenciated carcinoma with < 1% sarcomatoid	4	acidophilic / clear
	3	poorly differenciated carcinoma with 50% sarcomatoid	4	acidophilic / spindle cell
	4	poorly differenciated carcinoma with 90% sarcomatoid	4	acidophilic / spindle cell
	5	poorly differenciated carcinoma with 100% sarcomatoid	4	acidophilic / spindle cell
	6	poorly differenciated carcinoma with 90% sarcomatoid	4	acidophilic / spindle cell
	7	poorly differenciated carcinoma with 100% sarcomatoid	4	acidophilic / spindle cell
	8	poorly differenciated carcinoma with 50% sarcomatoid	4	acidophilic / spindle cell
	9	poorly differenciated carcinoma with 10% sarcomatoid	4	acidophilic / spindle cell
	10	poorly differenciated carcinoma	4	acidophilic
	11	poorly differenciated carcinoma	4	acidophilic
	12	poorly differenciated carcinoma with 50% sarcomatoid	4	acidophilic / spindle cell
**RCCPDX3**	0	clear cell with 20% sarcomatoid	3	acidophilic /clear
	4	clear cell	3	acidophilic / clear
	5	clear cell	4	acidophilic / clear
	6	clear cell	3	acidophilic / acinar
	7	clear cell	3	acidophilic / acinar
	8	clear cell with 10% sarcomatoid	4	acidophilic / spindle cell
	9	clear cell	3	acidophilic / acinar
	10	clear cell	3	acidophilic / acinar
	11	clear cell	3	acidophilic / acinar
	12	clear cell with <10% sarcomatoid	4	acidophilic / spindle cell
**RCCPDX4**	0	clear cell	2	clear/ acinar
	1	clear cell	3	acidophilic
	2	clear cell	3	acidophilic
	3	clear cell	3	acidophilic / acinar
**RCCPDX5**	0	clear cell	3	clear / acidophilic
	1	clear cell	2	clear
	2	clear cell	3	clear
**RCCPDX6**	0	mixed papillary 2 (50%) , clear (30%) with 20% sarcomatoid	3	acidophilic / clear / acinar / tubular/spindle cell
	2	mixed papillary 2, clear with sarcomatoid	3	acidophilic / clear / spindle cell
	3	clear cell	3	acidophilic / clear / acinar / tubular
**RCCPDX7**	0	clear cell	3	clear / acidophilic / acinar / tubular
	1	clear cell	3	acidophilic / clear
	2	clear cell	3	acidophilic / clear
	3	clear cell	3	clear / acidophilic
	4	clear cell	3	clear / acidophilic
	5	clear cell	3	clear / acidophilic
	6	clear cell	3	clear / acidophilic
	7	clear cell	4	acidophilic / clear
	8	clear cell	4	acidophilic / clear
	9	clear cell	3	clear / acidophilic
	10	clear cell	3	acidophilic / clear
	11	clear cell	4	acidophilic / clear
**RCCPDX8**	0	clear cell with 15% sarcomatoid	4	clear / acidophilic / spindle cell
	1	clear cell	3	acidophilic / clear
	2	clear cell	4	clear / acidophilic
	3	clear cell	2	acidophilic / acinar
	4	clear cell	4	acidophilic / clear
	5	clear cell	3	clear / acidophilic
	6	clear cell with 90% sarcomatoid	4	acidophilic / spindle cell
	7	clear cell	4	clear / acidophilic
	8	clear cell with 50% sarcomatoid	4	acidophilic / spindle cell
	9	clear cell with 50% sarcomatoid	4	acidophilic / spindle cell
	10	clear cell with 50% sarcomatoid	4	acidophilic / spindle cell
**RCCPDX9**	0	clear cell with 30% sarcomatoid	4	acidophilic / spindle cell
	1	clear cell	4	clear
	2	clear cell with rhabdoid	4	clear / acidophilic
	3	clear cell with 80% sarcomatoid	4	clear / acidophilic spindle cell
**RCCPDX10**	0	chromophobe with 80% sarcomatoid	4	acidophilic spindle cell 80% and chromophobe
	1	100% sarcomatoid	4	acidophilic / spindle cell
	2	100% sarcomatoid	4	acidophilic / spindle cell
	3	100% sarcomatoid	4	acidophilic / spindle cell
	4	100% sarcomatoid	4	acidophilic / spindle cell
	5	100% sarcomatoid	4	acidophilic / spindle cell
	6	100% sarcomatoid	4	acidophilic / spindle cell
	7	100% sarcomatoid	4	acidophilic / spindle cell
	8	100% sarcomatoid	4	acidophilic / spindle cell
**RCCPDX11**	0	clear cell with 50% sarcomatoid	4	clear / acidophilic / spindle cell
	1	clear cell with sarcomatoid	4	acidophilic / clear / spindle cell
	2	clear cell with sarcomatoid	4	acidophilic / clear / spindle cell
	3	clear cell with sarcomatoid	4	acidophilic / clear / spindle cell
**RCCPDX12**	0	clear cell	2	clear
	1	clear cell	3	clear / acinar
	2	clear cell	3	clear / acinar
**RCCPDX13**	0	clear cell with rhabdoid	4	acidophilic / clear
	1	clear cell with rhabdoid	2	acidophilic / clear
	2	clear cell with rhabdoid	4	acidophilic / clear
	3	clear cell with rhabdoid	4	acidophilic / clear / acinar
	4	clear cell with rhabdoid	4	acidophilic
	6	clear cell with rhabdoid	4	clear / acidophilic
	7	clear cell with 20% rhabdoid	4	acidophilic / clear / acinar
**RCCPDX14**	0	clear cell	3	clear / acidophilic / acinar / tubular
	1	clear cell	3	clear / acidophilic / acinar / tubular
	2	clear cell	2	clear / acidophilic / acinar
	3	clear cell	3	clear / acidophilic / acino / tubular
	4	clear cell	3	clear / acidophilic / acino / tubular
	5	clear cell	3	clear / acidophilic / acinar / tubular
**RCCPDX15**	0	clear cell with15% sarcomatoid	4	acidophilic / acinar / tubular / spindle cell
	1	clear cell	4	acidophilic / acinar
	2	clear cell	3	acidophilic / acinar and diffuse
	3	clear cell	4	acidophilic
	4	clear cell with 50% sarcomatoid	4	Acidophilic / spindle cell
	5	clear cell	4	acidophilic
	6	clear cell	3	acidophilic
	7	clear cell	4	acidophilic
	8	clear cell	4	acidophilic
	9	clear cell	4	acidophilic
	10	clear cell	4	acidophilic
**RCCPDX16**	0	clear cell	2	clear / acidophilic acinar
	1	clear cell	2	clear / acidophilic / acinar / tubular
	2	clear cell	2	clear / acidophilic / acinar
	3	clear cell	2	clear / acinar / tubular
	4	clear cell	2	clear / acidophilic
	5	clear cell	2	clear
	6	clear cell	2	clear / acidophilic
**RCCPDX17**	0	clear cell	2	clear / acidophilic / acinar / tubular
	1	clear cell	3	acidophilic and diffuse
	2	clear cell	3	acidophilic / acinar / tubular
	3	clear cell	2	clear / acidophilic / acinar / tubular
	4	clear cell	2	acidophilic / acinar / tubular
	5	clear cell	3	acidophilic / tubular
	6	clear cell	2	acidophilic / tubular
	7	clear cell	2	acidophilic / clear / acinar
	8	clear cell	2	acidophilic / acinar
	0	clear cell with 20% sarcomatoid	4	acidophilic / spindle cell
	1	clear cell with sarcomatoid	4	acidophilic / acinar and diffuse / spindle cell
	2	clear cell with sarcomatoid	4	acidophilic / acinar and diffuse / spindle cell
	3	clear cell with sarcomatoid	4	acidophilic / acinar and diffuse / spindle cell
	4	clear cell with 20% sarcomatoid	4	acidophilic / spindle cell
	5	clear cell	4	acidophilic
	6	clear cell with 20% sarcomatoid	4	acidophilic / spindle cell
	7	clear cell with 20% sarcomatoid	4	acidophilic / spindle cell
	8	clear cell with 20% sarcomatoid	4	acidophilic / spindle cell
	9	clear cell with 10% sarcomatoid	4	acidophilic / spindle cell
	10	clear cell with 10% sarcomatoid	4	acidophilic / spindle cell
**RCCPDX19**	0	clear cell rhabdoid (80%) with sarcomatoid 20%	4	acidophilic / spindle cell
	1	clear cell with sarcomatoid	4	acidophilic / acinar / spindle cell
	2	clear cell	4	acidophilic / acinar
	3	clear cell	3	acidophilic / acinar
	4	clear cell with 20% sarcomatoid	4	acidophilic / spindle cell
	5	clear cell with 100% sarcomatoid	4	acidophilic / spindle cell
	6	clear cell with 60% sarcomatoid	4	acidophilic / spindle cell
**RCCPDX20**	0	mixed papillary 2 and clear with 5% sarcomatoid	4	clear / acinar
	2	clear cell	2	clear / acidophilic / acinar / tubular
	3	clear cell with 20% sarcomatoid	4	acidophilic / acinar / spindle cell
	4	clear cell with 20% sarcomatoid	4	acidophilic / spindle cell
**RCCPDX21**	0	clear cell with 40% sarcomatoid	3	clear / tubular and diffuse acidophilic/ spindle cell
	1	clear cell	3	clear / acidophilic/ acinar and diffuse
	2	clear cell with sarcomatoid	4	clear / acidophilic / spindle cell
**RCCPDX22**	0	clear celL	3	clear
	1	clear cell	4	clear / acidophilic / acinar and diffuse
	2	clear cell	4	acidophilic / clear / acinar
	3	clear cell	4	acidophilic / acinar
	4	clear cell	4	clear / acidophilic
	5	clear cell	4	acidophilic / acinar
	6	clear cell	3	acidophilic / clear / acinar
	7	clear cell	3	acidophilic / clear / acinar
	8	clear cell	4	acidophilic / clear / acinar
**RCCPDX23**	0	clear cell	3	clear / acidophilic / acinar
	1	clear cell	3	acidophilic / clear / acinar
	2	clear cell	2	clear / acinar
	3	clear cell	2	clear / acinar
	4	clear cell	2	clear / acidophilic / acinar
	5	clear cell	3	clear / acidophilic / acinar
	6	clear cell	2	clear / acidophilic / acinar
	7	clear cell	3	clear / acidophilic (acinar)
	8	clear cell	2	acidophilic / clear (acinar)
	9	clear cell	3	acidophilic / clear (acinar)
	10	clear cell	2	clear / acidophilic (acinar)
**RCCPDX24**	0	unclassified with 100% sarcomatoid	4	acidophilic / spindle cell
	1	unclassified with sarcomatoid	4	acidophilic / spindle cell
	2	unclassified with sarcomatoid	4	acidophilic / spindle cell
	3	unclassified with sarcomatoid	4	acidophilic / spindle cell
	4	unclassified with sarcomatoid	4	acidophilic / spindle cell
	5	unclassified with sarcomatoid	4	acidophilic / spindle cell
	6	unclassified with sarcomatoid	4	acidophilic / spindle cell
	7	unclassified with sarcomatoid	4	acidophilic / spindle cell
	8	unclassified with sarcomatoid	4	acidophilic / spindle cell
	9	unclassified with sarcomatoid	4	acidophilic / spindle cell
	10	unclassified with sarcomatoid	4	acidophilic / spindle cell
**RCCPDX25**	0	clear cell	3	clear / acidophilic / tubular / acinar
	1	clear cell	3	clear
	2	clear cell	3	clear
	3	clear cell	3	clear
	4	clear cell	3	clear / acidophilic
	5	clear cell	4	clear / acidophilic
	6	clear cell	4	clear / acidophilic
	7	clear cell	4	clear / acidophilic
**RCCPDX26**	0	clear cell	3	clear
	1	clear cell	4	clear / acinar
	2	clear cell	3	clear / acidophilic / acinar
	3	clear cell	3	clear / acidophilic / acinar
	4	clear cell	3	clear / acidophilic / acinar
	5	clear cell	4	clear / acidophilic / acinar
	6	clear cell	4	clear / acidophilic / acinar
	7	clear cell	3	clear / acinar
**RCCPDX27**	0	medullary carcinoma	NA	acidophilic sheets
	1	medullary carcinoma	NA	acidophilic sheets
	2	medullary carcinoma	NA	acidophilic sheets
	3	medullary carcinoma	NA	acidophilic sheets
	4	medullary carcinoma	NA	acidophilic sheets
	5	medullary carcinoma	NA	acidophilic sheets
**RCCPDX28**	0	clear cell	4	acidophilic / clear
	1	clear cell	4	acidophilic / clear
	2	clear cell	4	acidophilic / clear
	3	clear cell	3	acidophilic / clear
**RCCPDX29**	0	papillary type 2	3	papillary
	1	papillary type 2	3	papillary
	2	papillary type 2	3	papillary
	3	papillary type 2	3	papillary
**RCCPDX30**	0	clear cell	4	clear / acidophilic / acinar / tubular
	1	clear cell	4	acidophilic / clear / acinar
	2	clear cell	4	acidophilic / clear / acinar
	3	clear cell	4	acidophilic / clear / acinar

**Figure 4:**
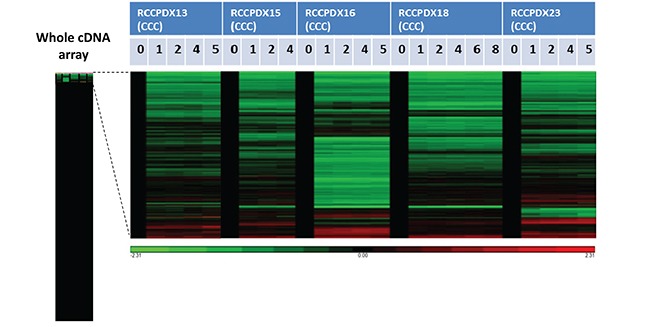
Affymetrix analysis of 5 RCCPDX tumors of the CCC subtype comparing the whole transcriptome of the original patient tumor (P0) to 3 to 5 passages (P1 to P8) in mice Gene expression in the various passages was compared to P0 that was set to 1 and appears in black. The left bar shows the whole analysis of cDNA array and the genes that were differentially expressed in passages compared to P0 are enlarged on the right. In green, genes that were overexpressed compared to P0 and in red, genes that were underexpressed compared to P0. Only a subset of genes were differentially expressed in passages compared to P0, and the differences were stable among passages for each RCCPDX (please see text for more details).

To determine whether serial xenografts keep molecular stability, we performed the analysis of the whole human transcriptome in a subset of RCCPDX models (RCCPDX13, 15, 16, 18 and 23) at P0 and at 3 to 5 subsequent passages in mice (P1 to P8, as indicated) (Figure 4). On a total of 20313 genes spotted on the human cDNA arrays there were between 116 (0.6%) and 399 (2.0%) genes differentially expressed depending on the RCCPDX model (data not shown). No specific molecular features could be deduced from the analysis of these differentially expressed genes. Analysis of the combined data showed that 33 differentially expressed genes were common among all RCCPX models tested 32 were down-regulated and 1 was up-regulated. However, no specific molecular features could be deduced from this restricted list (Table 4). Importantly, there was no change in gene expression among passages (Figure 4). The expression data files have been deposited in GEO, accession number GSE83820 (http://ncbi.nlm.nih.gov/geo/query/acc.cgi?acc=GSE83820).

**Table 4:** Common differentially expressed genes in the 5 RCCPDX analyzed by Affymetrix

**Up-regulated gene**
Hemoglobin, epsilon 1, mRNA
**Down-regulated genes**
Alpha-2-macroglobulin
Chromosome 13 open reading frame 15
Chromosome 16 open reading frame 54
Complement component 1, q subcomponent, B chain
Complement component 1, q subcomponent, C chain
CD163 molecule
CD52 molecule
CD93 molecule
Collagen, type XV, alpha 1
Endothelin receptor type B
EGF, latrophilin and seven transmembrane domain containing 1
Gtpase, IMAP family member 4
Gtpase, IMAP family member 6
G protein-coupled receptor 116
Major histocompatibility complex, class II, DQ alpha 1
Immunoglobulin heavy locus constant gamma 1 (G1m marker)
Lymphocyte cytosolic protein 2 (SH2 domain containing leukocyte protein of 76kda)
LIM domain binding 2
Immunoglobulin-like transcript 2b
Leukocyte immunoglobulin-like receptor, subfamily B (with TM and ITIM domains), member
Lysozyme (renal amyloidosis)
Myeloid cell nuclear differentiation antigen
Macrophage expressed 1
Membrane-spanning 4-domains, subfamily A, member 4
Membrane-spanning 4-domains, subfamily A, member 7
Platelet/endothelial cell adhesion molecule
Protein tyrosine phosphatase, receptor type, C
Regulator of G-protein signaling 1
Ribonuclease, rnase A family, k6
SAM domain, SH3 domain and nuclear localization signals 1
T-cell activation rho GTPase activating protein
TYRO protein tyrosine kinase binding protein

We further investigated whether genetic alterations were similar between the primary and subsequent grafted tumors through STR fingerprinting (Table 5) and VHL gene mutations analysis (Table 6). STR analysis was performed on DNA from all RCCPDX models at the indicated passages. VHL analysis was performed on DNA from the same RCCPDX models as specified above and at the indicated passages. STR analysis confirmed that xenografts came from the original patients' tumors, thus showing that there was no contamination within RCC tumors. We observed high rate of Y chromosome loss during passages compared to the original tumor P0, as reported in the literature [35]. Indeed, there were 22 RCCPDX models from patients with X and Y chromosomes present at P0, among which, in 11 cases, there was a loss of the Y chromosome during subsequent passages, i.e in half of the cases. There were some minor changes in the STR profile for some models, as expected when dealing with PDXs and as previously reported by other investigators, in RCC models and models derived from other tumor types (Table 5) [24, 25, 28]. Through direct sequencing of the 3 exons of the VHL gene, we detected mutations in 4 out of the 5 cases analyzed (80%). For all cases, identical mutations were observed between the primary tumor P0 and the subsequent passages (Table 6).

**Table 5:** Short tandem repeat fingerprinting

RCCPDX ID	AMEL	D10S1248	D12S391	D19S433	D1S1656	D22S1045	D2S1338	D2S441	D6S1043	TH01
RCCPDX1 /P0	X, Y	12	19 ; 22	13; 14	11; 14	13 ; 14	24	9,1	12 ; 19	8 ; 9,3
RCCPDX1 /P1	X	13 ; 14	19 ; 22	13; 14	11; 14	15	24	10 ; 11,3	12	8 ; 9,3
RCCPDX1 /P4	X	13 ; 14	19 ; 22	13; 14	11; 14	15	24	10 ; 11,3	12	8 ; 9,3
										
RCCPDX2 /P0	X, Y	13	19,1 ; 19,3	14 ; 15	11	15 ; 17	20 ; 25	14 ; 15	17	8 ; 9
RCCPDX2 /P1	X, Y	13, 14	19,1 ; 19,3	14 ; 15	11	17	20 ; 25	14 ; 15	12 ; 17	8 ; 9
RCCPDX2 /P4	X, Y	13	19,1 ; 19,3	14 ; 15	11	17	20 ; 25	14 ; 15	17	8 ; 9
										
RCCPDX3 /P0	X, Y	14 ; 15	17 ; 18	13 ; 14	12 ; 16	15	17 ; 20	10	11	6
RCCPDX3 /P1	X	14 ; 15	17 ; 18	13 ; 14	12 ; 16	15	17 ; 20	10	11	6
RCCPDX3 /P4	X	14 ; 15	17 ; 18	13 ; 14	12 ; 16	15	17 ; 20	10	11 ; 13	6
										
RCCPDX4 /P0	X	13 ; 14 ; 15	17 ; 22	14 ; 15	15 ; 18,3	15 ; 16	17 ; 19	10 ; 11	12 ; 17	8 ; 9
RCCPDX4 /P1	X	13 ; 14 ; 15	17 ; 22	14 ; 15	15 ; 18,3	15 ; 16	17 ; 19	10 ; 11	12 ; 17	8 ; 9
RCCPDX4 /P3	X	13 ; 14 ; 15	17 ; 22	14 ; 15	15 ; 18,3	15 ; 16	17 ; 19	10 ; 11	12 ; 17	8 ; 9
										
RCCPDX5 /P0	X, Y	13 ; 14 ; 15	15 ; 24	12 ; 16	12 ; 15	15 ; 16	17	11,3 ; 14	11 ; 18	9,3
RCCPDX5 /P2	X, Y	13 ; 14 ; 15	15 ; 24	12 ; 16	12 ; 15	15 ; 16	17	11,3 ; 14	11 ; 18	9,3
RCCPDX5 /P4	X, Y	13 ; 14 ; 15	15 ; 24	12 ; 16	12 ; 15	15 ; 16	17	11,3 ; 14	11 ; 18	9,3
										
RCCPDX6 /P0	X, Y	13 ; 14	17 ; 21	14	15 ; 17	11 ; 16	19 ; 24	10 ; 14	12 ; 17	9,3
RCCPDX6 /P1	X	13 ; 14	17 ; 21	14	17	16	19 ; 24	10 ; 14	12	9,3
RCCPDX6 /P3	X	13 ; 14	17 ; 21	14	17	16	19 ; 24	10 ; 14	12	9,3
RCCPDX7 /P0	X, Y	15 ; 16	17 ; 22	14 ; 15	16	14 ; 15	17 ; 23	11 ; 11,3	11 ; 14	8 ; 9,3
RCCPDX7 /P1	X	15	17	14 ; 15	16	14 ; 15	17	11 ; 11,3	11 ; 14	8 ; 9,3
RCCPDX7 /P4	X	15	17	14 ; 15	16	14 ; 15	17	11 ; 11,3	11 ; 14	8 ; 9,3
										
RCCPDX8 /P0	X, Y	13 ; 15	19 ; 23	12 ; 13	17,3 ; 18,3	15 ; 16	25	14	11	9
RCCPDX8 /P1	X	13 ; 15	19 ; 23	12 ; 13	17,3 ; 18,3	15 ; 16	25	14	11	9
RCCPDX8 /P4	X	13 ; 15	19 ; 23	12 ; 13	17,3 ; 18,3	15 ; 16	25	14	11	9
										
RCCPDX9 /P0	X, Y	13. 15	15 ; 21	13 ; 14	15	15	17 ; 26	11 ; 14	11 ; 20	6
RCCPDX9 /P1	X	13. 15	15 ; 21	13 ; 14	15	13 ; 15 ; 19	17 ; 26	11 ; 14	11 ; 22	6
RCCPDX9 /P4	X	13. 15	15 ; 21	13 ; 14	15	13 ; 15 ; 19	17 ; 26	11 ; 14	11 ; 22	6
										
RCCPDX10 /P0	X	13	23	14 ; 15	15	15	17	11	11	6 ; 8
RCCPDX10 /P1	X	13	23	14 ; 15	15	15	17	11	11	6 ; 8
RCCPDX10 /P4	X	13	23	14 ; 15	15	15	17	11	11	6 ; 8
										
RCCPDX11 /P0	X	16	17 ; 20	13,2 ; 14	15	14 ; 16	19 ; 24	11 ; 14	12 ; 14	6 ; 7
RCCPDX11 /P1	X	16	17 ; 20	13,2 ; 14	15	14 ; 16	24	11 ; 14	12	6 ; 7
RCCPDX11 /P3	X	16	17 ; 20	13,2 ; 14	15	14 ; 16	24	11 ; 14	12	6 ; 7
										
RCCPDX12 /P0	X	14 ; 15 ; 16	18 ; 22	14 ; 15	13	15 ; 16	19 ; 24	11	12 ; 13	6 ; 7
RCCPDX12 /P2	X	14 ; 15 ; 16	18 ; 22	14 ; 15	13	15 ; 16	19 ; 24	11	12 ; 13	6 ; 7
RCCPDX12 /P4	X	14 ; 15 ; 16	18 ; 22	14 ; 15	13	15 ; 16	19 ; 24	11	12 ; 13	6 ; 7
										
RCCPDX13 /P0	X;Y	13	17;19	14;15	16;17,3	14;16	23;25	11;15	11;13	7;8
RCCPDX13 /P1	X	13	17;19	14	16;17,3	14	23;25	11;15	13	7;8
RCCPDX13 /P4	X	13	17;19	14	16;17,3	14	23;25	11;15	13	7;8
										
RCCPDX14 /P0	X, Y	14	20	14,2 ; 16	17 ; 17,3	14	24 ; 25	10 ; 11	11 ; 19	9 ; 9,3
RCCPDX14 /P1	X, Y	14	20	14,2 ; 16	17 ; 17,3	14	24 ; 25	10 ; 11	11 ; 19	9 ; 9,3
RCCPDX14 /P4	X, Y	14	20	14,2 ; 16	17 ; 17,3	14	24 ; 25	10 ; 11	11 ; 19	9 ; 9,3
										
RCCPDX15 /P0	X;Y	13;15;16	16;19,3	11;12;14,3;16	16,3;17,3;18,3	16;17	17;19	9;10;11	10,3	8;9,3
RCCPDX15 /P1	X;Y	15;16;17	16;19,3	11;12;16	16,3;17,3;18,3;20,3	16;17	16;19	9;11	10,3	8;9,3
RCCPDX15 /P4	X;Y	15;16;17	16;18,3;19,3	11;12;16	16,3;17,3;18,3;20,3	16;17	16;19	9;10;11	10,3	8;9,3
										
RCCPDX16 /P0	X;Y	13;16	20;23	14	12;16,3	15;16	16;23	11	12;14	7
RCCPDX16 /P1	X	16	20;23	14	12	15;16	16	11	12	7
RCCPDX16 /P4	X	16	20;23	14	12	15;16	16	11	12	7
										
RCCPDX17 /P0	X, Y	16	21 ; 23	14 ; 15,2	12 ; 13	15	18 ; 23	11 ; 11,3	12 ; 13	9
RCCPDX17 /P1	X	16	21 ; 23	14 ; 15,2	12 ; 13	15	18 ; 23	11 ; 11,3	12	9
RCCPDX17 /P4	X	16	21 ; 23	14 ; 15,2	12 ; 13	15	18 ; 23	11 ; 11,3	12	9
										
RCCPDX18 /P0	X	14;15;16	19;26	12;13	12;16	15;16	19;25	10;11	12;13	9;10
RCCPDX18/P1	X	14;15;16	19;26	12;13	12;16	15	25	10;11	12;13	9;10
RCCPDX18/P3	X	14;15;16	19;26	12;13	12;16	15	25	10;11	12;13	9;10
										
RCCPDX19 /P0	X, Y	14	18 ; 22	14,2 ; 15	14 ; 16	16	17 ; 23	11 ; 14	11 ; 12	6
RCCPDX19 /P1	X	14	18	15	14 ; 16	16	17	11 ; 14	11 ; 12	6
RCCPDX19 /P4	X	14	18	15	14 ; 16	16	17	11 ; 14	11 ; 12	6
										
RCCPDX20 /P0	X, Y	13	17 ; 25	14 ; 17	15,3	11 ; 15	23	14	14 ; 18	7 ; 9
RCCPDX20 /P1	X, Y	13	17 ; 25	14 ; 17	15,3 ; 16	11 ; 15	23	14	14 ; 18	7 ; 9
RCCPDX20 /P4	X, Y	13	17 ; 25	14 ; 17	15,3 ; 16	11 ; 15	23	14	14 ; 18	7 ; 9
										
RCCPDX21 /P0	X, Y	14,16	18,3 ; 21	15	16 ; 19,3	16 ; 17	17	11 ; 14	11 ; 19	6 ; 9
RCCPDX21 /P1	X, Y	14,16	18,3 ; 21	15	16 ; 19,3	16 ; 17	17	11 ; 14	19	6 ; 9
RCCPDX21 /P2	X, Y	14,16	18,3 ; 21	15	16 ; 19,3	16 ; 17	17	11 ; 14	19	6 ; 9
										
RCCPDX22 /P0	X, Y	14 ; 15	16,3 ; 18,3	13 ; 15	16,3 ; 17,3	15, 16	17 ; 21	13 ; 14	11 ; 12	7 ; 9,3
RCCPDX22 /P1	X, Y	13 ; 14 ; 15	16,3 ; 18,3	13 ; 15	16,3 ; 17,3	15, 16	17 ; 21	12 ; 14	11 ; 12	7 ; 9,3
RCCPDX22 /P4	X, Y	14 ; 15	16,3 ; 17,3	13 ; 15	16,3 ; 17,3 ; 18,3	15, 16	17 ; 21	13 ; 14	12	7 ; 9,3
										
RCCPDX23 /P0	X;Y	14;16	18;21	13;14	14;16	16;18	19,3;25	10;14	11;13	9,3
RCCPDX23 /P1	X;Y	14;16	18	13;14	14;16	16	19,3;25	10;14	11;13	9,3
RCCPDX23 /P4	X	14;16	18	13;14	14;16	16	19,3;25	10;14	11;13	9,3
										
RCCPDX24 /P0	X	14	18 ; 20	14 ; 15	12 ; 17	16	17 ; 24	14	13 ; 14	8 ; 9,3
RCCPDX24 /P1	X	14	18 ; 20	14 ; 15	12 ; 17	16	17 ; 24	14	13 ; 14	8 ; 9,3
RCCPDX24 /P4	X	14	18 ; 20	14 ; 15	12 ; 17	16	17 ; 24	14	13 ; 14	8 ; 9,3
										
RCCPDX25 /P0	X	12 ; 14	21 ; 22	13 ; 15	12 ; 16	11 ; 14,3	19 ; 20	11	11	9 ; 9,3
RCCPDX25 /P1	X	14	21 ; 22	13 ; 15	16	15	19	11	11	9
RCCPDX25 /P4	X	12 ; 14	21 ; 22	13 ; 15	16	15	19	11	11	9
										
RCCPDX26 /P0	X, Y	14 ; 16	18 ; 21	13 ; 14	14 ; 18,3	13 ; 15	17 ; 25	11 ; 14	12 ; 19	9 ; 9,3
RCCPDX26 /P1	X, Y	14 ; 16		13		13 ; 15	17 ; 25	11 ; 14	12	
RCCPDX26 /P4	X, Y	14 ; 16	18 ; 21	13	14 ; 18,3	13 ; 15	17 ; 25	11 ; 14	12 ; 19	9 ; 9,3
										
RCCPDX27 /P0	X, Y	13 ; 16	15 ; 22	13 ; 15	14 ; 15	16 ; 17	17 ; 22	10 ; 11	20 ; 22	7 ; 9
RCCPDX27 /P1	X, Y	13. 16	15 ; 22	13 ; 15	14 ; 15	16	17 ; 22	10 ; 11	20 ; 22	7 ; 9
RCCPDX27 /P4	X, Y	13. 16	15 ; 22	13 ; 15	14 ; 15	16	17 ; 22	10 ; 11	20 ; 22	7 ; 9
										
RCCPDX28 /P0	X	13; 14	17 ; 24	15	16 ; 16,3	16	17 ; 23	11 ; 14	8 ; 11	9 ; 10
RCCPDX28 /P1	X	13 ; 14 ; 15	17 ; 24	15	16 ; 16,3	16	17 ; 23	11 ; 14	11	10
RCCPDX28 /P3	X	13 ; 15	17 ; 24	15	16 ; 16,3	16	17 ; 23	11 ; 14	8 ; 11	9 ; 10
										
RCCPDX29 /P0	X ; Y	14 ; 15	17	14 ; 15,2	11 ; 12	11 ; 14	17 ; 19	10 ; 11	12 ; 18	8 ; 9,3
RCCPDX29 /P1	X ; Y	14 ; 15	17	14 ; 15,2	11 ; 12	11 ; 14	17 ; 19	10 ; 11	12 ; 18	8 ; 9,3
RCCPDX29 /P3	X ; Y	14 ; 15	17	14 ; 15,2	11 ; 12	11 ; 14	17 ; 19	10 ; 11	12 ; 18	8 ; 9,3
										
RCCPDX30 /P0	X ; Y	12 ; 15	18 ; 21	13 ; 14	13 ; 14 ; 16,3	11 ; 16	15 ; 16	11 ; 13	14 ; 20	6
RCCPDX30 /P1	X ; Y	12 ; 15	18 ; 19 ; 21	12 ; 14	13 ; 15,3 ; 16,3	11 ; 16	15	12 ; 13	15 ; 21	6
RCCPDX30 /P4	X ; Y	12 ; 14 ; 15	18 ; 21	12 ; 13 ; 14	13 ; 15,3 ; 16,3	11 ; 16	15 ; 16	11 ; 13	15 ; 21	6

**Table 6:** Von Hippel-Lindau gene sequencing (VHL sequence accession number: NG_008212.3)

RCCPDX ID	Exon 1	Exon2	Exon3
RCCPDX13/P0	-	9945 dupT	-
RCCPDX13/1	-	9945 dupT	-
RCCPDX13/2	-	9945 dupT	-
RCCPDX13/4	-	9945 dupT	-
RCCPDX13/6	-	9945 dupT	-
			
RCCPDX15/P0	Del 5469-5474;Del 5477-5494	-	-
RCCPDX15/1	Del 5469-5474;Del 5477-5494	-	-
RCCPDX15/2	Del 5469-5474;Del 5477-5494	-	-
RCCPDX15/4	Del 5469-5474;Del 5477-5494	-	-
			
RCCPDX16/P0	-	-	Del 13238-13251
RCCPDX16/1	-	-	Del 13238-13251
RCCPDX16/2	-	-	Del 13238-13251
RCCPDX16/4	-	-	Del 13238-13251
RCCPDX16/5	-	-	Del 13238-13251
			
RCCPDX18/P0	-	9888 T>TA	-
RCCPDX18/1	-	9888 T>TA	-
RCCPDX18/2	-	9888 T>TA	-
RCCPDX18/4	-	9888 T>TA	-
RCCPDX18/6	-	9888 T>TA	-
RCCPDX18/8	-	9888 T>TA	-
			
RCCPDX23/P0	-	-	-
RCCPDX23/1	-	-	-
RCCPDX23/2	-	-	-
RCCPDX23/4	-	-	-
RCCPDX23/6	-	-	-

### Responses to therapeutic compounds

To assess whether RCCPDX models would reproduce the sensitivity to targeted therapies observed in the clinic, we tested the response to reference compounds *in vivo*.

**Figure 5:**
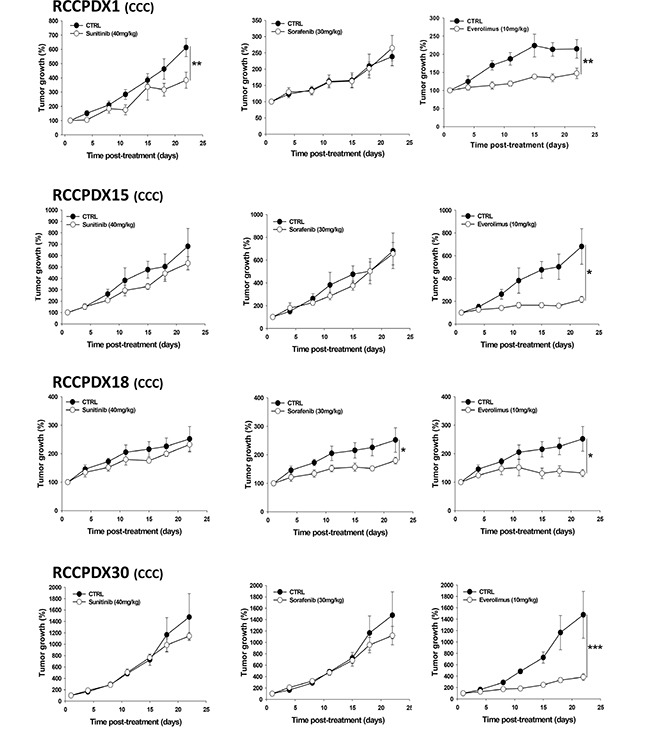
*In vivo* growth curves of 4 RCCPDX tumors of the CCC subtype treated with sunitinib, sorafenib or everolimus for the indicated time period Results are expressed in % from day 1 and as mean +/− sem, n=4 to 5 for each curve. *, P<0.05; **, P<0.01; ***, P<0.001 comparing treated to control groups. Note: mice were divided into four groups, the control and the treated groups i.e. one group for each compounds tested, except for RCCPDX1 where mice were divided into two groups, the control and the treated group for each compound tested.

We measured the response of 7 RCC models to sunitinib, sorafenib and everolimus. We observed a great variation in the profile of responses to the different therapies depending on the model considered (Figure 5 and Table 7). Tumors responded to sunitinib, the current first line therapy in 2 models, i.e 28% of cases, recapitulating what is observed in clinic. However, it is important to note that some tumors tested resistant to sunitinib were sensitive to either sorafenib or everolimus and one in the panel tested was sensitive to all three therapies. No complete response was observed in the course of these studies as it is the case in the large majority of clinical situations. It should be stressed that the original patients received treatment post- surgery in only rare cases in our RCCPDX models panel. For RCCPDX15 the corresponding patients was treated with nexavar, but he had severe side effects, and then temsirolimus; for RCCPDX18, the corresponding patient died before receiving sunitinib, and for RCCPDX 6, the corresponding patient received palliative care. The consequence of that was that there was only one RCCPDX model, RCCPDX10, that was derived from a patient who received sunitinib as first line therapy. The patient did not respond to the treatment and the same was observed in the RCCPDX model derived from his tumor (Table 7). Similarly, we observed sensitivity to sunitinib in a model derived from a node metastasis, exactly as the response of the patient (data not shown). With such a low number of cases, we could not assess the predictability value of the RCCPDX models generated.

**Table 7:** Additional patients' responses to targeted therapies

RCCPDX ID	Sunitinib	Sorafenib	Everolimus
**RCCPDX3**	NR	NR	R*
**RCCPDX4**	NR	R*	NR
**RCCPDX6**	R*	R**	R**
**RCCPDX10**	NR/PP	ND	ND

* P<0.05;

** P<0.01 from control.

R: Responder. NR: Non responder. PP: Predictive of the patient's therapeutic response. ND: not determined.

### Metastasis analysis

**Figure 6:**
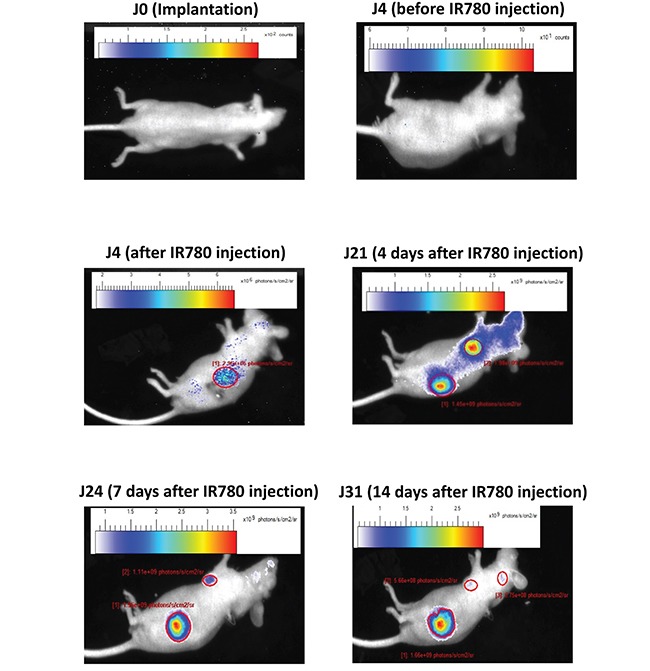
Metastasis analysis in an orthotopic model *In vivo* infrared imaging in RCCPDX20 after orthotopic implantation at different days before and after iv injection of the IR780 dye, showing primary tumors and metastasis development.

Primary tumors and metastasis were monitored during one month following implantation (Figure 6). No signal was observable before IR780 injection. In the model shown, RCCPDX20, primary tumor and lung metastasis were observed 3 weeks post-implantation; 4 weeks post-implantation, we also observed brain metastasis. These data indicate that during passages in mice, tumor tissues conserved their ability to invade, and at classical metastatic localizations.

## DISCUSSION

We xenografted in nude mice 336 RCC tumors of all subtypes and stages obtained from patients at the time of surgery from which we developed 30 models (P3 and above). It took up to 24 months to develop such model. We demonstrated that these models grow after both subcutaneous and orthotopic implantation, and are stable at the (i) histologic, (ii) genetic and (iii) molecular levels.

Histopathology analysis of all models showed that the histological features were preserved during passages as compared to the corresponding primary tumor, including tumor architecture, sarcomatoid components, cytology and Fuhrman grade. Similarly, at the genetic level, STR analysis of all models showed only minor changes, as well as a high rate of Y chromosome loss, as expected from previous studies [24, 25, 35, 36]. Molecular analysis using Affymetrix cDNA arrays performed on a subset of RCCPDX models obtained at different times also revealed the stability of the models compared to the corresponding primary tumor. The analysis of the differentially expressed genes did not allow the definition of a particular molecular signature, that could for example influence engraftment. Such investigations will necessitate the analysis of a large number of tumors that successfully grow in mice vs. tumors that do not grow, and compare them eventually to previous studies where molecular data and analysis are available. This was not the scope of the present work.

Higher stage, grade and sarcomatoid differentiation were among the parameters we studied that favor engraftment. Here, we obtained an engraftment success rate of 8.9% by xenografting tumors of all sub-types and at all stages and grades, and all tumors were established as transplantable tumors for at least 13 passages. In previous papers from other investigators, in which authors xenografted from 2 to 94 tumors including in some instances metastasis, the engraftment rates ranged between 37 and 100% [23–34]. This was of course dependent on the size of the cohort, the number of passages in mice, and on the characteristics of the implanted tumors (pTNM stage, tumor size, Fuhrman grade, primary vs. metastatic tissue, unilateral or bilateral cancer and focal or multifocal tumor). For example, in Angevin et al. publication [30], tumorigenicity was correlated with the metastatic phenotype of the tumor (54% success rate) and with reduced survival of patients; in Sivanand et al. publication [28] metastatic tissues engrafted at higher rate than those from primary tissues, and the stability of the engraftment correlated with decreased patient survival.

In the present study, clinical history and follow-up were available for all patients. Primary tumors and corresponding models were characterized at various biological levels and shown to be stable. Importantly, nude mice bearing some PDX tumors were specifically treated with current therapies (sunitinib, sorafenib and everolimus) to assess their sensitivity and the concordance with the clinical situation. When challenged to current targeted therapies, each model behaved differently depending on the respective therapy. Importantly, when available, the models responded to the therapy exactly as the patient from whom the xenograft was derived. However, in a clinical point of view, all patients are treated the same way since no predictive biomarkers have yet been validated for these drugs as well as for new potential therapeutic compounds currently under clinical evaluation. This lack of biomarkers restricts our ability to tailor specific drugs to patients and might be considered as the most important barrier for a better clinical response.

It should be stressed, however, that in the present study only one model, and another but derived from a metastatic site (not included in the RCCPDX panel presented here) were available to assess whether the models generated may have predictivity value, i.e similar or identical therapeutic response than the parental tumor. The results obtained with these two models are therefore not conclusive regarding predictivity of the therapeutic response. However, these models reproduce the sensitivity to targeted therapies observed in the clinic, thus closely mimicking human RCC.

Thus, this panel of RCCPDX models should be valuable for studying the mechanisms of therapy-induced resistance, and for the design of prognostic tools based on molecular signatures of the tumors, which should help to better design therapy tailored to the patient. This is clearly of great value to identify predictive biomarkers of therapeutic response and of therapy-induced resistance. Moreover, these PDX models could be used for screening any new emerging treatment for RCC, as well as for repositioning existing drugs, allowing for a rapid and cost efficient screening of response biomarkers that will be the base of personalized medicine.

RCC tumor grafts have been successfully generated by some independent groups by xenografting primary and/or metastastic tissues [23–34]. The PDX generated were comparable to parental tumors, at least with regard to the parameters analyzed including histology, genetic and molecular features. When available, metastatic and drug responsiveness recapitulated what is observed in clinic. The comparison between our work and that of these other groups may be quite difficult since the panel of tumors xenografted differs from one study to another as well as the number of passages, from 1 to 50, and the date of establishment during the last 30 years. However, each of these panels is useful and of great importance for translational research in the RCC field.

In conclusion, we have developed realistic preclinical models of RCC that will greatly accelerate the development of new therapeutic compounds and the elucidation of response and resistance mechanisms to current therapeutics. These models are difficult to develop, although sarcomatoid components of the tumors seem to greatly enhance the take rate, a feature that could not be specified when dealing with low numbers of PDX models. To our knowledge, our study constitutes one of the largest panel of preclinical PDX models for RCC. This panel will be useful for both patient prognosis and drug response since they recapitulate parental tumors histologically, genetically and molecularly. We can thus generate precise and reliable data, directly available for clinical applications, and this constitutes the first step to personalized medicine.

## MATERIALS AND METHODS

### Animals

4-week old male Nu/Nu athymic mice were purchased from Charles River (L'Arbresle, France). Mice were housed in ventilated carousel racks and provided with sterile food and drink water. All the mouse experiments reported herein were approved by Animal Housing and Experiment Board of the French government.

### Patients and tumor processing and grafting

Fresh samples were obtained from 336 human RCC tumors between 2007 and 2014 (Table 1 and Table 2). All patients provided written informed consent. Patient material was de-identified according to clinical processes and French law regulations for patient information and consent. After surgery, tissue specimens were immediately transferred on ice in DMEM medium additioned with penicillin/streptomycine to the animal facility. Tumors were dissected, washed in DMEM medium, cut into 5 mm^3^ pieces and grafted subcutaneously in 5 mice under general isoflurane gaseous anaesthesia. All this procedure was performed in sterile conditions and in less than 30 min post-surgery. For each tumor, some pieces of tissue were snap-frozen in liquid nitrogen for genetic and molecular characterization, others formalin-fixed for histological or immunohistological analysis and the rest keep frozen in FBS/DMSO mixture (90/10%) used for new passages (P) in mice. In addition, pieces of corresponding normal tissues harvested at the edge of the tumors at the time of surgery were snap-frozen in liquid nitrogen and others formalin-fixed for tumor/normal tissues comparison studies. The study was conducted in accordance with the Declaration of Helsinski.

### Tumor passaging and storage

Once the grafted tumors reached 500-1000 mm^3^, mice were subjected to general anaesthesia provided as stated in the appendix and tumors were dissected under sterile conditions. Tumors were then cut into small pieces of 5 mm^3^ and washed in PBS. Again, as stated in the appendix for the primary tumors (P0), the pieces were divided into 4 parts, one of them used for the subsequent passages. To date, the tumors that developed in mice have been serially passages up to 13 passages (P13).

### Orthotopic tumor implantation

Mice were placed on the right lateral side under general anaesthesia as stated above. A skin incision was made in the left flank to localize the left kidney. The renal capsule was then incised and a small piece of tumor obtained from subcutaneous implantation was then placed under the capsule. The abdominal wall was then closed with suture.

### Histology

For all RCCPDX models, primary and passaged tumors preserved in formalin were paraffin-embedded and process into 5 μm thick cuts and placed on glass slides. Hematoxylin and eosin (H&E) staining and slides analysis were performed by an experienced uropathologist.

### Transcriptome analysis

Total RNA from patients' primary tumors and from corresponding tumors at passage ranging from P1 to P8 was obtained using Qiagen columns according to manufacturer's protocol. The concentration and integrity/purity of each RNA sample were measured using RNA 6000 LabChip kit (Agilent) and the Agilent 2100 bioanalyzer. U133 Plus 2.0 array containing 54,624 probe sets excluding the AFFY quality control probe sets representing 20313 human genes (Affymetrix, Santa Clara, CA, USA). The Transcriptome analysis was performed by Firalis SAS (Huningue, France), a biotech specialized in biomarkers identification on 100 ng of total RNA that were amplified and labeled according to the Affymetrix protocol. The RMA data were reported as log2-transformed intensities. Descriptive statistics antilog intensities across all tumors were used. Prefiltering excluded all probe sets with Affy QC. The expression values for each individual passaged tumors was normalized separately on primary tumor expression values. Log2 transformation of fold changes (FC) was used. In order to check the quality of the individual microarrays the intensity distribution of all samples were calculated and compared. Exploration analysis included principal component analysis, hierarchical clustering and heatmap visualization.

### Short tandem repeat analysis

DNA from patients' primary tumors and from corresponding tumors at passage ranging from P1 to P4 was obtained by phenol/chloroform extraction A nanodrop ND-1000 spectrophotometer (Thermo scientific, Illkirch, France) was used to determine DNA concentration and purity. DNA samples were subjected to short tandem repeat (STR) DNA fingerprinting using the AuthentiFiler PCR amplification Kit (Life technologies, Saint Aubin, France) that amplifies 9 unique STR loci (8 that comprise tetranucleotide repeat units and one locus trinucleotide) and the Amelogenin gender-determining marker, according to manufacturer instructions. PCR products were separated by capillary electrophoresis on a genetic analyzer ABI PRISM 3100 and results analyzed using the GeneMapper software.

### Von Hippel-Lindau gene sequencing

The 3 exons encoding the VHL gene were amplified by polymerase chain reaction (PCR) using specific primers pairs The high fidelity KAPA Taq DNA polymerase (Clinisciences, Nanterre, France) was used and PCR products were purified using nucleospin PCR clean-up columns (Macherey-Nagel, Hoerdt, France). Both directions sequencing as well as sequence alignment and comparison to the reference sequence was performed by Millegen (Labège, France), and GATC biotech (Cologne, Germany).

### Treatment with reference compounds

For each serie, once tumor volume reached a palpable size (around 100 mm^3^), mice were randomly divided into different groups, control (diluent) and treated groups, as indicated in the corresponding Figure legend. Mice were treated per os with diluent (cremophor 10%, DMSO 5% in PBS) or sunitinib, sorafenib or everolimus (Euromedex, Souffelweyersheim, France). Sunitinib (40 mg/kg) was administered 3 times/week for 3 weeks. Sorafenib (30 mg/kg) and everolimus (10 mg/kg) were administered 5 times per week for 3 weeks. Tumor growth was measured using a caliper as previously detailed [10].

### Tumor and metastasis imagery

To image tumors and metastasis we used the Heptamethine cyanine dye IR-780 iodide which accumulates in tumor cells [37]. IR-780 0.2 mg/kg, 200 μl) was injected ip 24-48h before NIR imaging coupled to X-ray which was performed using a multimodality imaging system for small animal (Biospace photon imager, Institut Pluridisciplinaire Hubert Curien, Strasbourg).

### Statistical analysis

All values are expressed as mean ± s.e.m. Statistical analysis was performed when appropriate using Student's *t* test, one-way or two-way ANOVA followed by the Student-Newman-Keul's test for multiple comparisons.

For cDNA arrays on Affymetrix, a 2 way ANOVA considering tissue and passage (P0 to P8) as factors and Post-Hoc tests (contrasts) for P0 vs P1, and P1 vs P2, P1 vs P4, P1 vs P5, P1 vs P6, and P1 vs P8, were used.

A *P* < 0.05 was considered significant (Benjamini-Hochberg).

## Abbreviations

CCC: clear cell renal cell carcinoma
PDX: patient-derived tumor xenograft
VHL: von Hippel-Lindau tumor suppressor gene
